# The complete dorsal structure is formed from only the blastocoel roof of *Xenopus* blastula: insight into the gastrulation movement evolutionarily conserved among chordates

**DOI:** 10.1007/s00427-023-00701-1

**Published:** 2023-03-18

**Authors:** Yuki Sato, Izumi Narasaki, Takuya Kunimoto, Yuki Moriyama, Chikara Hashimoto

**Affiliations:** 1 JT Biohistory Research Hall, 1-1 Murasaki-Cho, Takatsuki Osaka, 569-1125 Japan; 2 Department of Biology, Graduate School of Science, Osaka University, Suita, Japan; 3 Faculty of Science and Engineering, Chuo University, Hachioji, Japan

**Keywords:** Gastrulation, Chordate, *Xenopus*, Epiblast, Yolk

## Abstract

Gastrulation is a critical event whose molecular mechanisms are thought to be conserved among vertebrates. However, the morphological movement during gastrulation appears to be divergent across species, making it difficult to discuss the evolution of the process. Previously, we proposed a novel amphibian gastrulation model, the “subduction and zippering (S&Z) model”. In this model, the organizer and the prospective neuroectoderm are originally localized in the blastula’s blastocoel roof, and these embryonic regions move downward to make physical contact of their inner surfaces with each other at the dorsal marginal zone. The developmental stage when contact between the head organizer and the anterior-most neuroectoderm is established is called “anterior contact establishment (ACE).” After ACE, the A-P body axis elongates posteriorly. According to this model, the body axis is derived from limited regions of the dorsal marginal zone at ACE. To investigate this possibility, we conducted stepwise tissue deletions using *Xenopus laevis* embryos and revealed that the dorsal one-third of the marginal zone had the ability to form the complete dorsal structure by itself. Furthermore, a blastocoel roof explant of the blastula, which should contain the organizer and the prospective neuroectoderm in the S&Z model, autonomously underwent gastrulation and formed the complete dorsal structure. Collectively, these results are consistent with the S&Z gastrulation model and identify the embryonic region sufficient for construction of the complete dorsal structure. Finally, by comparing amphibian gastrulation to gastrulation of protochordates and amniotes, we discuss the gastrulation movement evolutionarily conserved among chordates.

**Supplementary Information:**

The online version contains supplementary material available at 10.1007/s00427-023-00701-1.

## Introduction

Gastrulation is a critical developmental event for producing three germ layers, three body axes, and the central nervous system in the embryo. While the molecular mechanisms involved in gastrulation are thought to be conserved among vertebrates, the morphogenetic movement is described as divergent. For example, the amphibian dorsal blastopore lip and the chick Hensen’s node express the same genes, such as *goosecoid* and *noggin* (Cho et al. 1991; Izpisúa-Belmonte et al. 1993; Smith and Harland 1992; Connolly et al. 1997), and both tissues show a potent secondary axis-inducing ability upon transplantation (Spemann and Mangold 1924; Leikola, 1976). Therefore, these tissues are considered the equivalent structure, termed the organizer. However, whereas the amphibian dorsal blastopore lip is thought to form a notochord anteriorly by animalward migration on the inner surface of the blastocoel roof, the chick Hensen’s node migrates and forms a notochord posteriorly. Thus, it has been considered so far that the direction of notochord formation is opposite between amphibians and avians, and therefore, it is difficult to discuss the evolution of the morphogenetic movement.

**Fig. 1:**
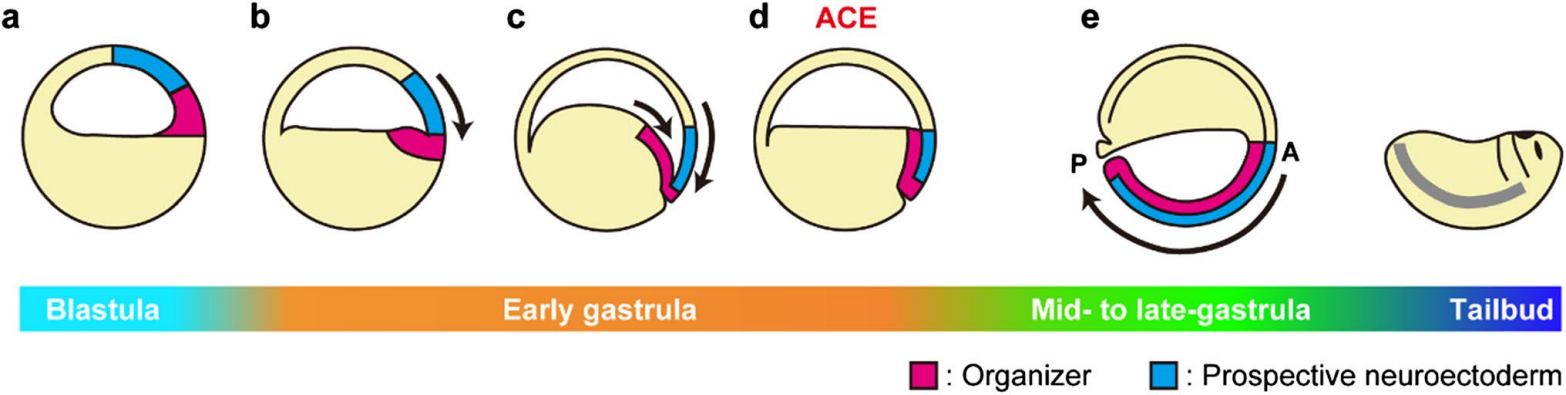
Schematic drawing of S&Z gastrulation model. **a** The organizer and the prospective neuroectoderm are originally established tandemly at the blastocoel roof of the blastula. **b** The organizer and the prospective neuroectoderm move downward. The organizer finally localizes at the blastocoel floor. **c** The prospective neuroectoderm progresses with downward movement. Accompanying that, the organizer is forcibly dragged down and makes a trench, which is termed “subduction movement.” Then, the organizer and the prospective neuroectoderm make physical contact from posterior (vegetal) toward anterior (equatorial), termed “zippering movement.” **d** As a result of subduction and zippering (S&Z) movement, “Anterior Contact Establishment (ACE)” occurs at the dorsal marginal zone of early gastrula. (**e**) The axial structure is formed toward the posterior during gastrulation. The red region and the blue region indicate the organizer and the prospective neuroectoderm, respectively

Previously, we proposed an amphibian gastrulation model (Fig. 1; Koide et al. 2002; Yanagi et al. 2015) which is totally different from the conventional model drawn in a wide variety of both scientific papers and textbooks. Briefly, the organizer is originally located in the blastocoel roof of the blastula adjacent to the prospective neuroectoderm around the animal pole (Fig. 1a). Both tissues move downward to the equatorial region by epiboly movement (Keller 1978). Eventually, the organizer tissue is located at the dorsal portion of the blastocoel floor by the onset of gastrulation (Fig. 1b). Subsequently, the prospective neuroectoderm and the organizer become closer to establish physical contact with each other by subduction and zippering (S&Z) movement, which is probably performed by the combination of both epiboly (Keller 1978) and vegetal rotation (Winklbauer and Schürfeld 1999) (Fig. 1c). The developmental stage when the physical contact between the anterior-most neuroectoderm and the head organizer occurs is called “anterior contact establishment (ACE)” (Fig. 1d), at which the whole region of neural tissues is determined. In the case of *Xenopus laevis*, ACE occurs in the first 3 h of the whole 20-h process of gastrulation at 13 °C. After ACE, the head region is fixed at the dorsal equatorial region, and the notochord is formed posteriorly through the vegetal pole (Fig. 1e). Consistent with this model, the body axis was formed from the dorsal marginal zone to the vegetal side of the embryo when the *Xenopus* eggs were embedded into gelatin to prevent the free rotation due to the shift of the center of gravity (Yanagi et al. 2015). Moreover, the cell tracing by transplantation of the GFP-labeled dorsal marginal zone at ACE into a non-labeled ACE embryo revealed that the sliding between the mesoderm and the neuroectoderm proposed in the conventional model did not occur, supporting the S&Z gastrulation model (Koide et al. 2002).

According to the S&Z model, ACE occurs at the dorsal marginal zone in a quite early step of gastrulation. Therefore, the complete neural tissues can be formed without the blastocoel roof of the gastrula. To test this, we removed the blastocoel roof of the embryo at ACE in various amphibian species, including anuran and urodele species, and allowed it to develop. We found that all of the operated embryos underwent gastrulation and developed dorsal structures consisting of the head, elongated trunk, and tail, but did not develop the ventral epidermis (Yanagi et al. 2015). The dorsal structures are equivalent to the structures of the secondary body axis formed upon the organizer transplantation. Thus, this experiment indicated that the blastocoel roof of the ACE embryo contributes only to the ventral epidermis, which supports the S&Z model. This result prompted us to investigate where the region corresponding to the complete dorsal structure in the early *Xenopus* embryo was located based on the S&Z model.

Thus, in this study, stepwise tissue deletions were conducted to identify the embryonic region corresponding to the complete dorsal structure in *X. laevis*. The results revealed that the blastocoel roof, the ventral two-thirds of the marginal zone, and most of the endoderm in the ACE embryo were not required for the formation of the complete dorsal structure. Rather, a dorsal one-third of the marginal zone at ACE was sufficient for the formation of this structure. Further analysis revealed that the blastocoel roof of the blastula also constructed the complete dorsal structure by itself. Both the dorsal one-third of the marginal zone at ACE and the blastocoel roof of the blastula contain the whole region of the organizer and the prospective neuroectoderm in the S&Z model, verifying the adequacy of the S&Z gastrulation model. We discuss the evolution of gastrulation movement among chordates from the perspective of the S&Z model.

## Materials and methods

### Frogs

*Xenopus laevis* purchased from Watanabe Zoushoku (Hyogo, Japan) were maintained in JT Biohistory Research Hall (Osaka, Japan). Embryos were obtained by in vitro fertilization. The jelly coat was removed chemically with 3% cysteine (pH 8.0) solution. Embryos were kept in 0.1 × Barth’s solution (8.8 mM NaCl, 0.1 mM KCl, 0.24 mM NaHCO_3_, 0.082 mM MgSO_4_·7H_2_O, 0.033 mM Ca(NO_3_)_2_·4H_2_O, 0.041 mM CaCl_2_·2H_2_O, 0.5 mM HEPES, pH 7.6) until they reached the desired stage according to Nieuwkoop and Faber (1994). The animal experiments were conducted according to JT Biohistory Research Hall Regulations on Animal Experimentation.

*Odorrana supranarina* was maintained by the Institute for Amphibian Biology (Hiroshima University) through the National Bio-Resource Project of MEXT, Japan. Embryos of *O. supranarina* were obtained by natural mating. The embryos were kept in 0.1 × Steinberg’s solution (58 mM NaCl, 0.67 mM KCl, 0.44 mM Ca(NO_3_)_2_, 1.3 mM MgSO_4_, 4.6 mM Tris, pH 7.8) at 18 °C.

### Tissue deletion experiments and whole-mount in situ hybridization

The blastocoel roof and marginal zone were removed at the level of the blastocoel floor with fine forceps at the ACE gastrula stage and cultured in 1x Barth’s solution until they reached the indicated stage. The yolky endoderm was removed with fine forceps from the opened blastocoel roof of ACE embryos in 1x Barth’s solution for one hour. The resultant yolky endoderm-less embryos were transferred into fresh 0.1 × Barth’s solution until they reached the indicated stage. The dorsal marginal zone was cut out and cultured in 1x Barth’s solution until it reached the indicated stage. The blastocoel roof of the blastula was excised at stage 8 with fine forceps and cultured until it reached the indicated stage in 1x Barth’s solution. In situ hybridizations were performed as previously described (Hemmati-Brivanlou et al. 1990).

### Labeling injection and transplantation of the dorsal marginal zone

For in vitro mRNA synthesis, pCS2-*mGFP* (containing a Gap43 membrane signal) was linearized by NotI and transcribed with SP6 RNA polymerase using the Ambion mMessage mMachine kit. Four hundred picograms of thus-synthesized *mGFP* mRNA in 4 nL was injected into dorsal or ventral blastomeres at the 4-cell stage. When the pigmentation at the blastopore had just appeared, labeled embryos were selected. The dorsal marginal zone was excised from a donor ACE embryo and transplanted into the ventral side of a host ACE embryo and cultured in 1x Barth’s solution. Grafted embryos were transferred into fresh 0.1 × Barth’s solution and allowed to develop until they reached the indicated stage.

## Results

### Blastocoel roof and ventral two-thirds of the marginal zone at ACE were dispensable for the formation of the complete dorsal structure

Since ACE occurs at the dorsal equator of the early gastrula and then, the A-P axis is formed posteriorly by convergent extension movement in the S&Z model, it could be possible to think that the complete dorsal structure is constructed from a restricted region at the dorsal equator of the ACE embryo, which corresponds to the conjugant of the organizer (red) and the overlying ectoderm (blue) in Fig. 1d. To investigate this possibility, we tried to narrow down the embryonic region(s) necessary for the formation of the complete dorsal structure.

**Fig. 2:**
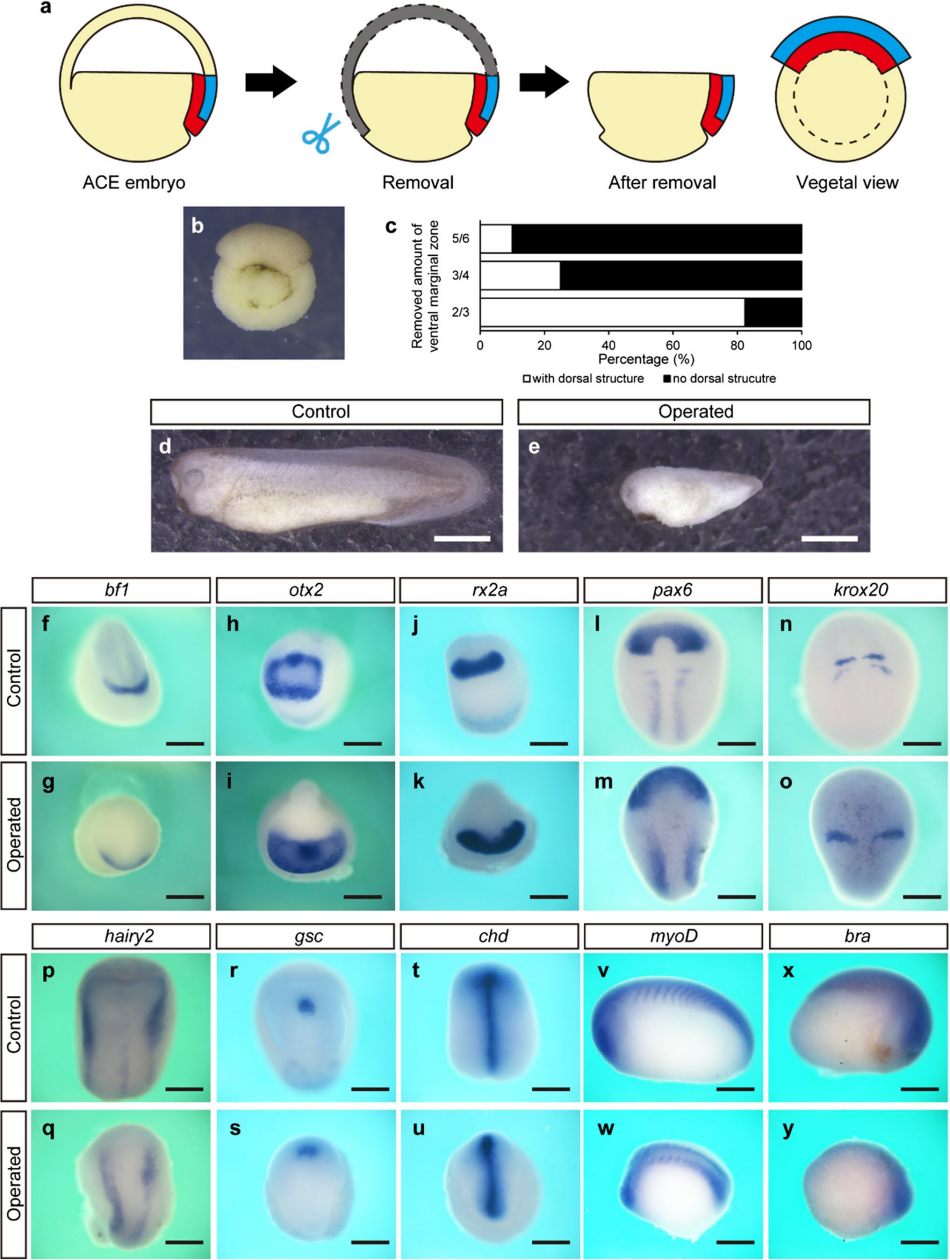
ACE embryo lacking blastocoel roof and ventral two-thirds of the marginal zone developed the complete dorsal structures. **a** Schematic drawing of removal of the blastocoel roof and the ventral marginal zone at ACE. **b** The ACE embryo after removal of the blastocoel roof and ventral marginal zone. **c** The percentage of the embryo with dorsal structure after the removal of the indicated amount of the vegetal marginal zone at ACE. **d**, **e** The tailbud embryos developed from control and operated ACE embryo. Scale bars, 1 mm. **f**–**y** In situ hybridization of control and operated embryos. Scale bars, 500 µm. The expression of *bf1* (**f**, **g**), *otx2* (**h**, **i**), *rx2a* (**j**, **k**), *pax6* (**l**, **m**), *krox20* (**n**, **o**), *hairy2* (**p**, **q**), *gsc* (**r**, **s**), and *chd* (**t**, **u**) in the neurula stage. The expression of *myoD* (**v**, **w**) and *bra* (**x**, **y**) in the tailbud stage

The blastocoel roof at ACE has been shown to be dispensable for the formation of the complete dorsal structure via gastrulation (Yanagi et al. 2015). Hence, the vegetal half of the ACE embryo should contain the corresponding embryonic region for the complete dorsal structure. Since the dorsal structures are suggested to be developed from the dorsal marginal zone of the ACE embryo in the S&Z model (Fig. 1d, e), the ventral marginal zone could be dispensable. To test this possibility, we conducted stepwise deletions of the ventral marginal zone from the blastocoel roof-less ACE embryo (Fig. 2a, b). When the ventral half of the marginal zone was removed at ACE, all of the operated embryos underwent gastrulation and successfully formed the dorsal structures without ventral epidermis at the tailbud stage. Furthermore, removing the ventral two-thirds of the marginal zone at ACE resulted in formation of the dorsal structures with apparent optic vehicles and cement gland (though it showed a short A-P axis) in 82.4% (14/17) of the operated embryos (Fig. 2c–e; Supplementary movie 1). On the other hand, when The ventral three-fourths or five-sixths of the marginal zone was removed from ACE embryos, the efficiency of the dorsal structure formation was greatly reduced to 25% (5/20) or 10% (1/10), respectively (Fig. 2c). These results suggest that the dorsal one-third of the marginal zone is required for the dorsal structure formation. To check whether the developed dorsal structures were properly patterned, whole-mount in situ hybridization (WISH) of several marker genes was conducted. Compared to the control neurulae, the operated neurulae which were constructed from the ACE embryos lacking both the blastocoel roof and the ventral two-thirds of the marginal zone showed proper expression patterns of the marker genes of forebrain (*bf1*, *otx2*), retina (*rx2a*, *pax6*), hindbrain (*krox20*), neural crest (*hairy2*), prechordal plate (*gsc*), and notochord (*chd*) (Fig. 2f–u). This indicated that the operated embryos had the properly organized neural and axial mesodermal tissues. Moreover, the operated tailbud embryos showed the same expression patterns of the marker genes of somite (*myoD*) and tailbud (*bra*) as the control embryos (Fig. 2v–y), indicating that somitic mesoderm was properly developed in the operated embryos. These results suggest that the blastocoel roof and the ventral two-thirds of the marginal zone at ACE are dispensable for the complete dorsal structure formation.

### Formation of the complete dorsal structure did not require most of the yolky endodermal tissue at ACE

**Fig. 3:**
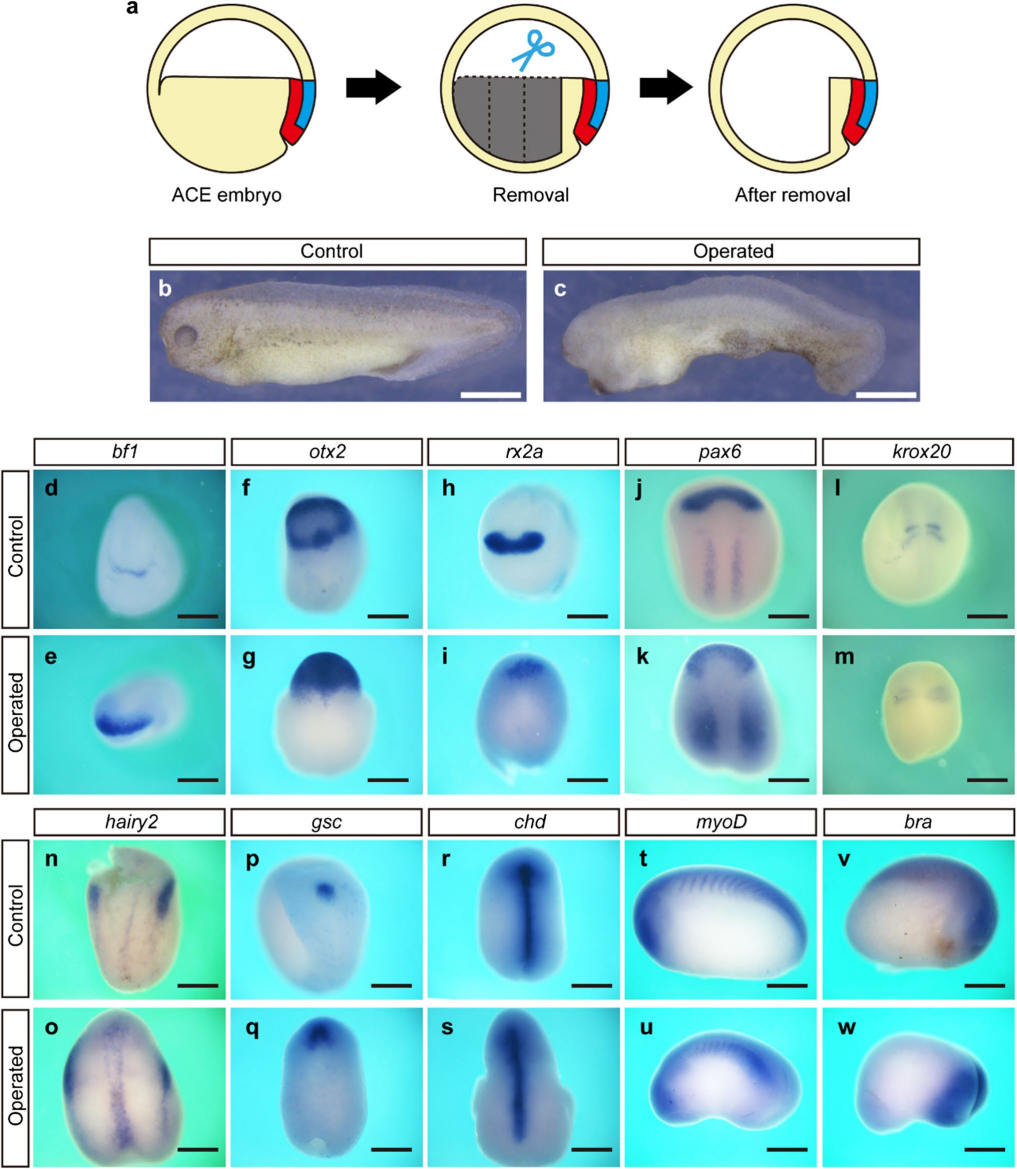
ACE embryo lacking yolky endoderm developed the complete dorsal structures. **a** Schematic drawing of the yolky endoderm removal at ACE. **b**, **c** The tailbud embryos developed from control and operated ACE embryo. Scale bars, 1 mm. **d**–**w** In situ hybridization of control and operated embryos. Scale bars, 500 µm. The expression of *bf1* (**d**, **e**), *otx2* (**f**, **g**), *rx2a* (**h**, **i**), *pax6* (**j**, **k**), *krox20* (**l**, **m**), *hairy2* (**n**, **o**), *gsc* (**p**, **q**), and *chd* (**r**, **s**) in the neurula stage. The expression of *myoD* (**t**, **u**) and *bra* (**v**, **w**) in the tailbud stage

It is known that the organizer marker genes, such as *goosecoid* and *chordin*, are expressed in about two to three cell layers of the most dorsal inner tissue at early gastrula (Cho et al. 1991; Kuroda et al. 2004). If the formation of the complete dorsal structure requires only the dorsal one-third of the marginal zone containing the organizer, most of the inner tissue (yolky endoderm) may be dispensable. Therefore, we removed most of the yolky endodermal tissues (ventral three-quarters in length) from the ACE embryo and allowed it to undergo development (Fig. 3a). As expected, the operated endoderm-less embryos developed to the tailbud stage had dorsal structures containing a complete head, elongated trunk, and tail, although it had lost most of the endodermal yolk mass (Fig. 3b, c; Supplementary movie 2). The neurulae developed from the endoderm-less ACE embryo properly expressed the neural and the axial mesodermal tissue marker genes (Fig. 3d–s). Furthermore, the operated embryos at the tailbud stage properly expressed *myoD* and *bra* (Fig. 3t–w). These results indicate that the dorsal structures developed from the endoderm-less embryo have proper neural and axial/somitic mesodermal tissues. Thus, most of the yolky endoderm should be dispensable for the development of the complete dorsal structure.

### The dorsal one-third of the marginal zone at ACE was sufficient for development of the complete dorsal structure

**Fig. 4:**
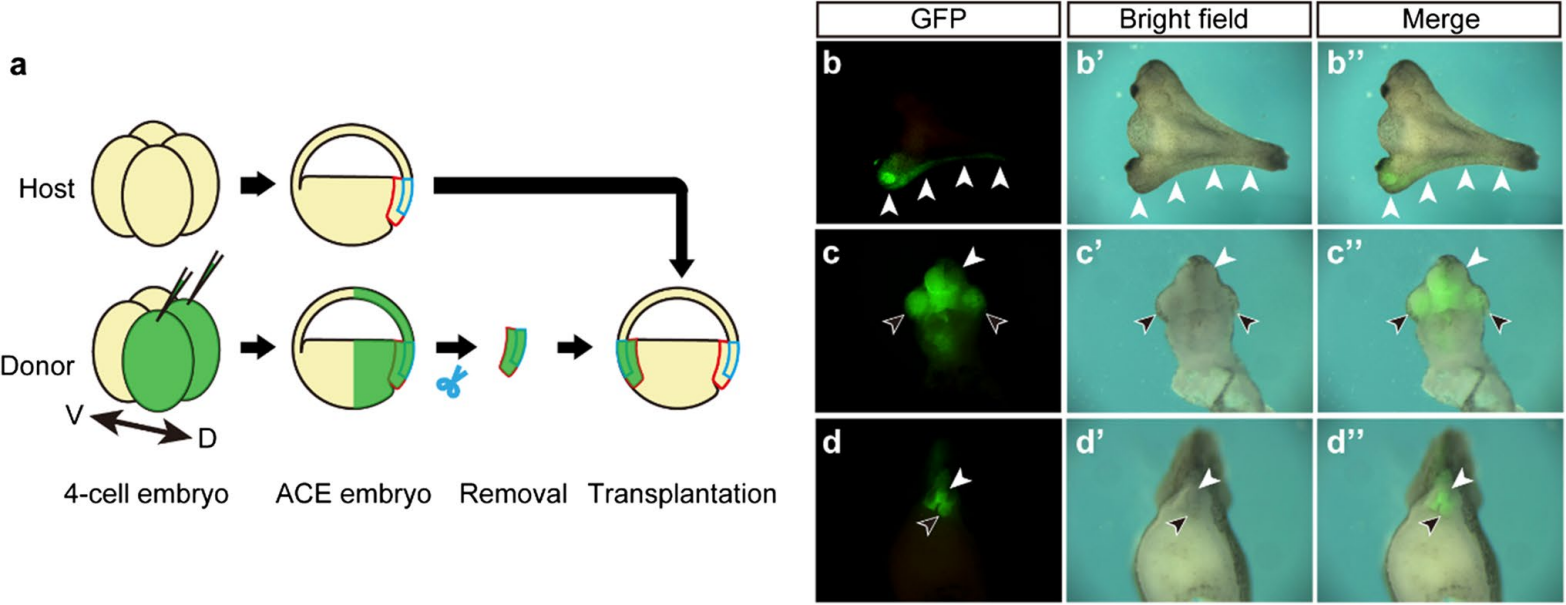
Transplantation of mGFP-labeled dorsal marginal zone at ACE into the ventral side of non-labeled ACE embryo. **a** Schematic drawing of the transplantation assay. **b** The whole-mount view of operated embryo at the tailbud stage. White arrowheads, mGFP-labeled secondary body axis. **c** The transverse section of head in the secondary body axis. White arrowhead, neural tube. Black arrowheads, eyes. **d** The transverse section of trunk region in the secondary body axis. White arrowhead, neural tube. Black arrowhead, notochord

The above results indicate that the blastocoel roof, ventral two-thirds of the marginal zone, and most of the yolky endoderm of the ACE embryo are not required for the formation of the complete dorsal structure. Therefore, only the dorsal one-third of the marginal zone at ACE, which may contain the organizer and the overlying ectoderm, is suggested to be sufficient for the construction of the complete dorsal structure. To examine this possibility, *membrane GFP* (*mGFP*) mRNA was injected into the two dorsal blastomeres at the 4-cell stage, and the mGFP-labeled dorsal one-third of the marginal zone at ACE was transplanted into the ventral side of another non-labeled ACE embryo (Fig. 4a). As a result, a labeled secondary axis was constructed at the ventral side of the host embryo (Fig. 4b). The transverse sections of the secondary axis contained the brain, eyes, neural tube, notochord, and medial somites. All of these tissues in the secondary axis expressed mGFP (Fig. 4c, d). In the conventional gastrulation model, the Spemann organizer migrates toward the animal pole on the inner surface of the blastocoel roof and induces neural fates, which should result in making the brain, the eyes, and the neural tube in the secondary axis that are negative for mGFP. Therefore, our result suggests that the secondary axis was formed from the transplanted graft, not by induction from the transplant. However, it was unclear whether the secondary body axis was constructed only from the transplanted cells. To clarify this, we conducted transplantation of non-labeled dorsal one-third of the marginal zone at ACE into the ventral side of labeled host ACE embryo (Suppl. Fig. 1a). The transplanted graft formed the secondary axis possessing completely non-labeled head and tail, whereas its trunk showed a salt-and-pepper-like distribution of labeled host cells and non-labeled donor cells (Suppl. Fig. 1b, c). The fact that the trunk regions of the secondary axis had some labeled host cells suggested that the trunk elongation might occur by convergent extension movement with surrounding host cells. When the ventral two-thirds of the marginal zone was removed from the ACE embryo, it developed a shorter A-P axis than control embryos (Fig. 2c, d). Thus, the more ventral marginal zone at ACE is removed, the shorter an A-P axis is formed, suggesting that the surrounding marginal tissue is required for the elongation of the trunk region but not necessary for the construction of the dorsal structures.

These results suggest the possibility that the solo culture of the dorsal one-third of the marginal zone at ACE could form the complete dorsal structure. Then, the dorsal one-third of the marginal zone at ACE, which consisted of two-to-three cell layers of inner tissues and the overlying ectoderm, was explanted and allowed to develop until the tailbud stage (Suppl. Fig. 2a, b). In the conventional gastrulation model, the blastocoel roof of the gastrula is required for the establishment for neural tissues. Interestingly, however, the tiny explant developed into an embryoid which seemed to have optic vehicles, cement gland, and tailbud, though it showed a shorter A-P axis than control embryos (Suppl. Fig. 2c, d; Supplementary movie 3), suggesting that the complete dorsal structure could be constructed in the embryoids. WISH analysis confirmed that the developing explants equivalent to the neurula stage showed the expression of forebrain (*bf1*, *otx2*), retina (*rx2a*, *pax6*), hindbrain (*krox20*), neural crest (*hairy2*), prechordal plate (*gsc*), and notochord (*chd*) marker genes (Fig. S2e–t). In addition, the embryoids equivalent to the tailbud stage expressed *myoD* and *bra* (Fig. S2u–x). Taken altogether, these results indicate that the dorsal one-third of the marginal zone at ACE is able to construct the complete dorsal structures by itself.

### Blastocoel roof of the blastula was able to develop the complete dorsal structure

**Fig. 5:**
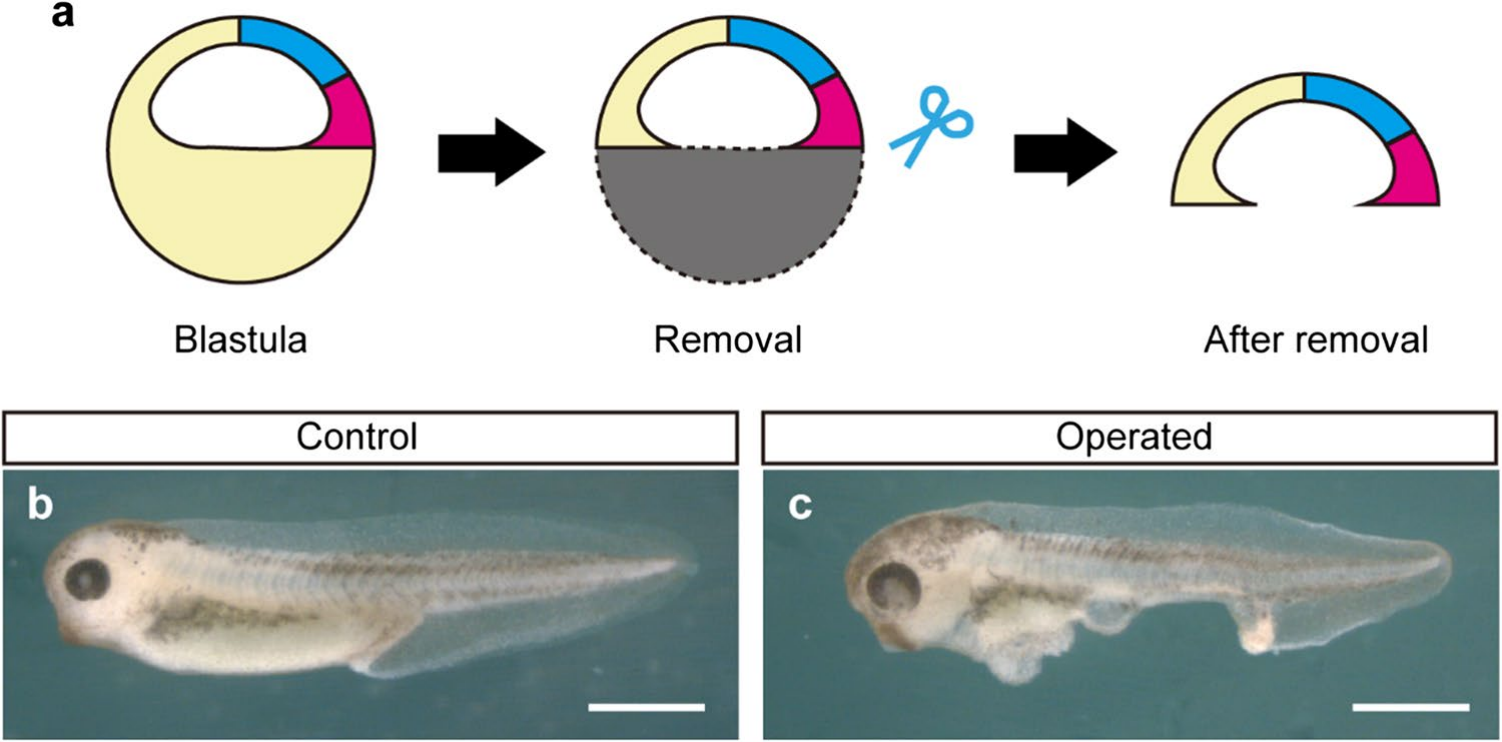
The explant of blastocoel roof of blastula developed an embryo only lacking yolk mass. **a** Schematic drawing of the blastocoel roof explant of blastula. **b**, **c** The tailbud embryos developed from control and operated blastocoel roof explant of blastula. Scale bars, 1 mm

In the S&Z model, the organizer and the prospective neuroectoderm are thought to be initially localized at the blastocoel roof of the blastula and to move downward by the time of ACE to establish the physical contact (Fig. 1a, b; Yanagi et al. 2015). Then, since the development of the complete dorsal structure requires only the dorsal one-third of the marginal zone at ACE, the blastocoel roof of the blastula where the region was originally localized might be sufficient to form the complete dorsal structure. To test this idea, the blastocoel roof of the blastula at stage 8 was explanted and allowed to develop until the tailbud stage (Fig. 5a). After the excision, the transplant rapidly closed the wound site. At the stage equivalent to ACE, a small blastopore appeared and the subsequent gastrulation processes progressed. The neural plate and the neural fold were also properly formed. At last, surprisingly, the explants developed into almost-complete tailbud embryos only lacking the ventral yolk mass (Fig. 5b, c: Supplementary movie 4). The resultant phenotype looks quite similar to that developed from the endoderm-less ACE embryo (Fig. 3b, c). Thus, these results indicated that the blastocoel roof of the blastula, which includes the organizer and the overlying ectoderm of the ACE embryo, contains all of the embryonic regions corresponding to the complete dorsal structure.

## Discussion

In the S&Z gastrulation model, the organizer and the prospective neuroectoderm are originally established in the blastocoel roof of blastula embryos, and these embryonic regions move downward to get into contact with each other by their inner surfaces at the dorsal marginal zone in the ACE embryo (Fig. 1). In this study, we found that the dorsal one-third of the marginal zone at ACE is sufficient for the construction of the complete dorsal structure, indicating that this small embryonic region should contain not only the organizer and the prospective neuroectoderm but also the precursors of somitic mesoderm, neural crest, and other dorsal components. Since organizer marker genes such as *goosecoid* and *chordin* have been shown to be expressed in around the dorsal one-third of the marginal zone at early gastrula (Cho et al. 1991; Sasai et al. 1994), the entire organizer tissue may be necessary for the formation of the complete dorsal structure. In addition, when the mGFP-labeled dorsal one-third of the marginal zone at ACE was transplanted into the ventral side of a non-labeled ACE embryo, almost all the structures of the secondary body axis were labeled (Fig. 4). According to the conventional gastrulation model, the organizer migrates on the inner surface of the blastocoel roof and induces neural fate, which should result in the construction of neural structures by non-labeled host cells in the transplantation assay. However, the current result showed that not only the organizer tissues but also the neural and neural crest derivatives are constructed by the transplants, indicating that this secondary axial structure is not the result of induction by the Spemann organizer (Spemann and Mangold 1924) but rather is already determined within the transplanted dorsal one-third of the marginal zone at ACE as described in the S&Z model (Fig. 1; Yanagi et al. 2015). In addition, the head and tail of the secondary axis were constructed from the transplanted dorsal one-third of the marginal zone, but the trunk region was formed by both transplant and host cells (Fig. 4, Suppl. Fig. 1). Therefore, it is suggested that the head and tail structures are constructed from the tissues within the dorsal one-third of the marginal zone, and the elongated trunk is constructed from both the dorsal one-third of the marginal zone and the neighboring marginal tissues by convergent extension movement.

It is noteworthy that the Keller sandwich explant constructed from ACE embryos developed the organized neural tissues expressing *otx2* and *krox20* in order (Koide et al. 2002). Since the Keller sandwich is the back-to-back conjugate of dorsal ectodermal tissues (Keller and Danilchik 1988), it contains the prospective neuroectoderm but not the underlying organizer or other mesodermal/endodermal tissues, in principle. Therefore, it is suggested that the neural domains such as forebrain, midbrain, and hindbrain have already been specified within the prospective neuroectoderm by the time of ACE. However, the Keller sandwich can never form more complicated and detailed neural structures, indicating that the construction of the complete dorsal structures requires the organizer even after ACE for purposes other than the induction and the initial patterning of neural tissues. For example, the formation of two separate eyes requires the underlying axial mesoderm (Li et al. 1997), and the prechordal mesoderm is known to be necessary for hypothalamus formation (Yamaguti et al. 2005; Xie and Dorsky 2017).

**Fig. 6:**
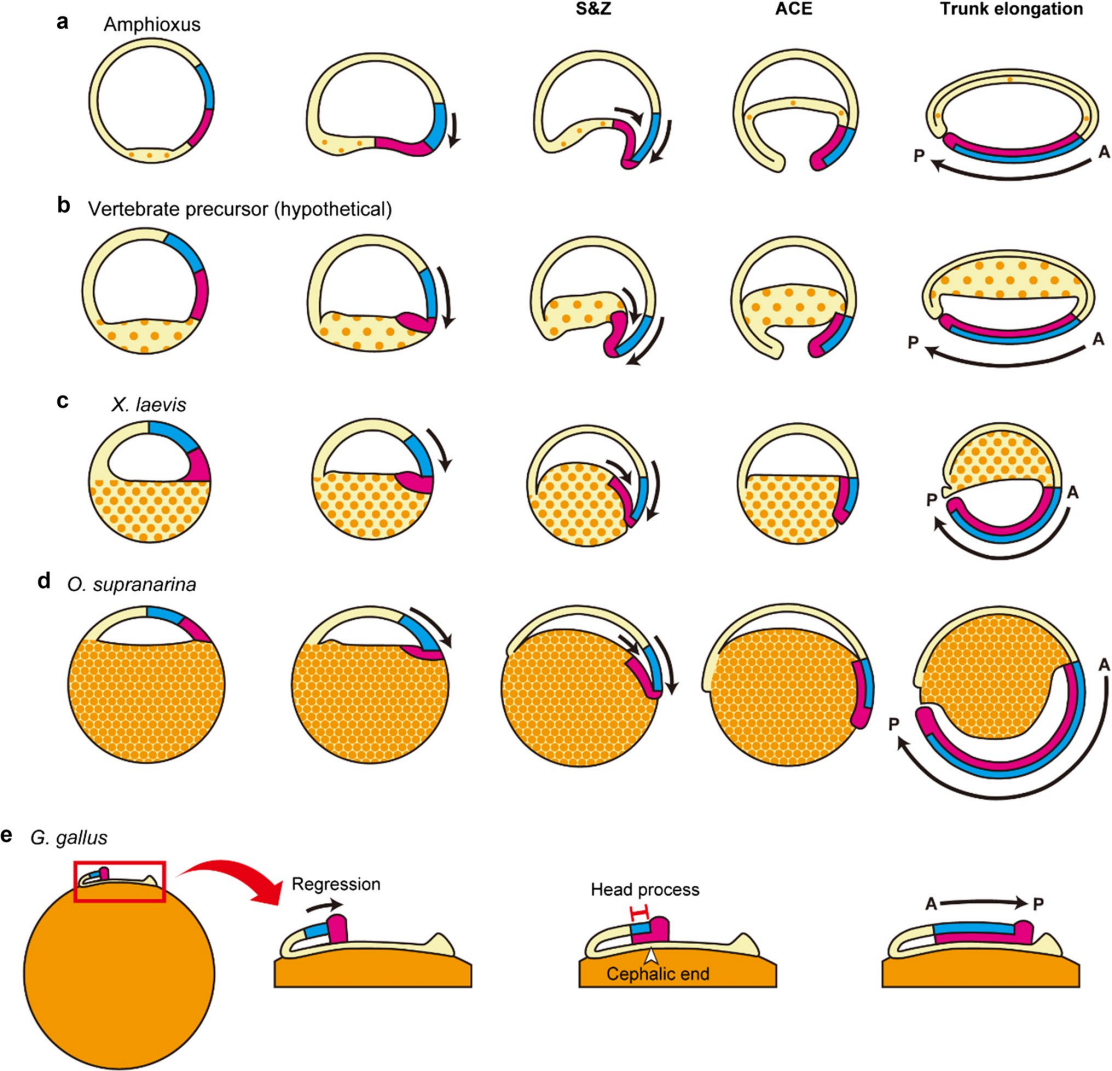
S&Z movement is conserved, whereas the amount and the location of yolk are varied, among chordates. **a** Amphioxus shows a hollow globular embryo which possesses only a small amount of yolk, but ACE has occurred by the simple invagination with little involution, which corresponds to S&Z movement. Thereafter, the A-P axis is elongated posteriorly. **b** Though vertebrate precursor might acquire the yolk on the vegetal side of the embryo, the gastrulation movement could be conserved. **c** Amphibians such as *Xenopus laevis* also store a certain amount of yolk within their dividing vegetal hemisphere. **d** *Odorrana supranarina* has a larger amount of yolk, which impedes cleavage, but the gastrulation movement, in which ACE occurs at the dorsal marginal zone and elongates the A-P axis posteriorly, is indeed confirmed. **e** In avian development such as that of *Gallus gallus*, the anterior end of the primitive streak regresses after the streak reaches its full length. The regression might lead to physical contact between the organizer and the prospective neuroectoderm, which could be referred to as the head process. The morphogenetic movement is similar to S&Z movement. Red region, the organizer. Blue region, the prospective neuroectoderm. Orange, yolk

When the blastocoel roof of the blastula was explanted, the wound healed quickly and the explant underwent gastrulation movement and eventually developed an embryo lacking only a yolk mass (Fig. 5). Consistent with the current results, it was reported that *Xenopus* fertilized eggs in which 40% of the vegetal cytoplasm was removed after the cortical rotation could develop into embryos lacking a yolk mass (Sakai 1996). Collectively, all of these findings suggest that the development of the complete dorsal structure does not require the vegetal part of the embryo before gastrulation in *Xenopus*. The wound-healed blastula blastocoel roof explant may show a hollow globular structure which is similar to the blastula of a basal chordate (amphioxus). The amphioxus blastula forms the blastopore, and the presumptive mesoderm which expresses the organizer genes such as *goosecoid* and *chordin* undergoes simple invagination with little involution to make a physical contact with the ectodermal region between their respective inner surfaces (Fig. 6a; Zhang et al. 1997; Yu et al. 2007). At this standpoint, the gastrulation movement of amphioxus appears to be equivalent to the amphibian S&Z movement. Thus, the wound-healed explant of the blastocoel roof of *Xenopus* blastula might mimic the situation of the amphioxus blastula, and if so, it could be thought that the gastrulation movement is conserved from amphioxus to amphibians. One of the differences between blastula embryos of amphioxus and amphibians is thought to be the amount of yolk. The vegetal side of the *Xenopus* oocyte has numerous yolk platelets, which are highly conserved membrane-bound organelles storing yolk proteins such as the derivatives of Vitellogenin (Danilchik and Gerhart 1987), and the yolk platelets segregate to the cleaved blastomeres resulting in the larger amount of yolk platelets in the vegetal blastomeres. In addition, the developing gut of amphibians, which is derived from the vegetal blastomere, consumes the numerous yolk platelets to feed the embryo like a yolk sac in later developmental stages (Jorgensen et al. 2009). Therefore, it could be thought that the vegetal half of the amphibian blastula corresponds to amniote yolk (Fig. 6c). On the other hand, the amphioxus embryo contains only a small amount of yolk (Fig. 6a). Therefore, it could be thought that the vertebrate precursors increase the amount of yolk on the vegetal side of the embryo to store more nutrients for development (Fig. 6b). From this point of view, the *Xenopus* embryo might be regarded as the embryo of a basal chordate with a certain amount of yolk in its vegetal side but the S&Z gastrulation movement is basically conserved. Based on this idea, the endoderm-less ACE embryo and the blastocoel roof explant from the blastula in this study could be regarded as essentially the same: the difference between the two operated embryos is whether the yolk was removed before or after ACE, and both contain the regions required for the formation of the complete dorsal structure. Therefore, these operated embryos developed similar tailbud embryos lacking a yolk mass (Figs. 3 and 5).

While the eggs of basal chordates and amphibians undergo holoblastic cleavage, the eggs of amniotes such as birds and reptiles show meroblastic cleavage because they harbor a huge amount of condensed yolk which impedes cleavages. Therefore, it is difficult to compare the gastrulation movement between amphibians and amniotes directly. However, the egg of *Odorrana supranarina*, which is a species of frog inhabiting Ishigaki Island and Iriomote Island in Japan, has a condensed yolk and shows meroblastic cleavage on the animal pole side similar to avian meroblastic discoidal cleavage. Therefore, *O. supranarina* could be considered an intermediate species between general amphibians and amniotes. When the fertilized egg was embedded into gelatin to prevent the rotation following the shift of the center of gravity as previously described (Yanagi et al. 2015), the dorsal structures were formed on the vegetal side in *O. supranarina* (Supplementary movie 5), as in *Xenopus* embryos. When the cortical rotation was prevented by treatments such as UV irradiation or nocodazole treatment, the dorsal structure could not be constructed (Gerhart et al 1989). Therefore, the cortical rotation was not disturbed by embedding into gelatin, and the elongation of the body axis to the vegetal side of the embryo could be considered a physiological and not an artificial process, which is conserved among amphibians, even in *O. supranarina*. Thus, it is suggested that the amphibian embryos showing meroblastic cleavage also follow the S&Z gastrulation model even though these amphibians have an apparent condensed yolk in their eggs (Fig. 6d). From this perspective, we also think that a movement similar to the amphibian S&Z movement can be found in the chick developmental process. After the primitive streak has reached its full length, the anterior end (Hensen’s node) begins to regress, leaving in its trail a structure commonly referred to as the head process (Nicolet 1971; Wakely and England 1979). Hence, it could be thought that the prospective head neural tissue, which is localized anterior to Hensen’s node, also moves posteriorly with the regression and contributes to the formation of the head process (Fig. 6e). If so, these relative movements to form the head process look like the S&Z movement of amphibian gastrulation. Therefore, from the comparison of the gastrulation movement of an amphibian to that of a basal chordate and an amniote, it is suggested that the fundamental manners of gastrulation are conserved among chordates though individual processes are different from species to species: the prospective neuroectoderm and the organizer make a physical contact by their inner surfaces and elongate the A-P axis posteriorly (Fig. 6).

Wnt/β-catenin signaling is an evolutionarily conserved signaling pathway regulating numerous biological processes (Clevers and Nusse 2012). When the *Xenopus* egg is fertilized, the cortical rotation occurs and the maternal dorsal determinants including Disheveled (Dsh) and GSK3-binding protein (GBP) are actively transported to the presumptive dorsal region along the microtubule bundles by binding to kinesin (Weaver et al. 2003). The translocated dorsal determinants then lead to the nuclear accumulation of β-catenin, which induces gene expression including expression of the Nodal-related factors (McKendry et al. 1997; Takahashi et al. 2000). Of note, the maternal transmembrane protein Huluwa, which also localizes at the vegetal pole of the oocyte and dorsally translocates upon the cortical rotation, was recently reported to induce the nuclear accumulation of β-catenin at the dorsal region in a Wnt protein-independent manner (Yan et al. 2018). Furthermore, macropinocytosis and the formation of lysosomes are suggested to be required for β-catenin signaling in the dorsal region of *Xenopus* early embryos (Tejeda-Muñoz and De Robertis 2022). The Spemann organizer has been reported to be established by the cooperation of β-catenin and Nodal signaling, which is conserved among the early chordate embryos (Kozmikova and Kozmik 2020). Consistent with these reports, the nuclear accumulation of β-catenin at the dorsal blastocoel roof of the early blastula has been reported (Schohl and Fagotto 2002). Therefore, we suggest that Dsh, GBP, Hwa and other molecules translocate to the dorsal region of the embryo upon the cortical rotation to lead to the establishment of the organizer at the blastocoel roof of the blastula, and then, it moves downward to the dorsal marginal zone of the early gastrula.

In the early chick development, the midgut remains open and connects to the yolk sac, while the dorsal structures including head, trunk, and tail are definitely constructed from the epiblast. As described above, in *Xenopus* development, the vegetal half of the blastula, which contributes to the gut, corresponds to the yolk of amniotes, and the blastocoel roof of the blastula develops the complete dorsal structure. In addition, it is known that the *Xenopus* animal cap cells can be converted into different types of cells such as endodermal, mesodermal, epithelial, and neural cells by several growth factors (Asashima et al. 1990; Ariizumi et al. 1991; Kengaku and Okamoto 1993). Therefore, though the animal cap of the *Xenopus* blastula is frequently referred to as undifferentiated ectoderm, it could be equivalent to the epiblast in amniotes rather than ectoderm. Thus, we proposed that all chordate embryos could be divided into two fundamental portions: (1) the embryonic tissues, which are the blastocoel roof of the blastula in amphibians and the epiblast in amniotes, and (2) the extraembryonic tissues acquired by increasing the amount of yolk in the evolution of chordates, which are the vegetal half of the blastula in amphibians and the yolk in amniotes.

## Supplementary Information

Below is the link to the electronic supplementary material.

Supplementary file1 (DOCX 2038 KB)

Supplementary file2 (MP4 2697 KB)

Supplementary file3 (MP4 12510 KB)

Supplementary file4 (MP4 26472 KB)

Supplementary file5 (MP4 6520 KB)

Supplementary file6 (MP4 11402 KB)

## Data Availability

All data generated or analyzed during this study are included in this published article and its supplementary information files.
