# Supplementary material for: The complete dorsal structure is formed from only the blastocoel roof of Xenopus blastula: insight into the gastrulation movement evolutionarily conserved among chordates
Source: Dev Genes Evol. 2023 Mar 18;233(1):1–12. doi: 10.1007/s00427-023-00701-1 (PMC10239386; doi:10.1007/s00427-023-00701-1)
Supplement: Supplementary file 1 — Supplementary file1 (DOCX 2038 KB) [file 427_2023_701_MOESM1_ESM.docx]

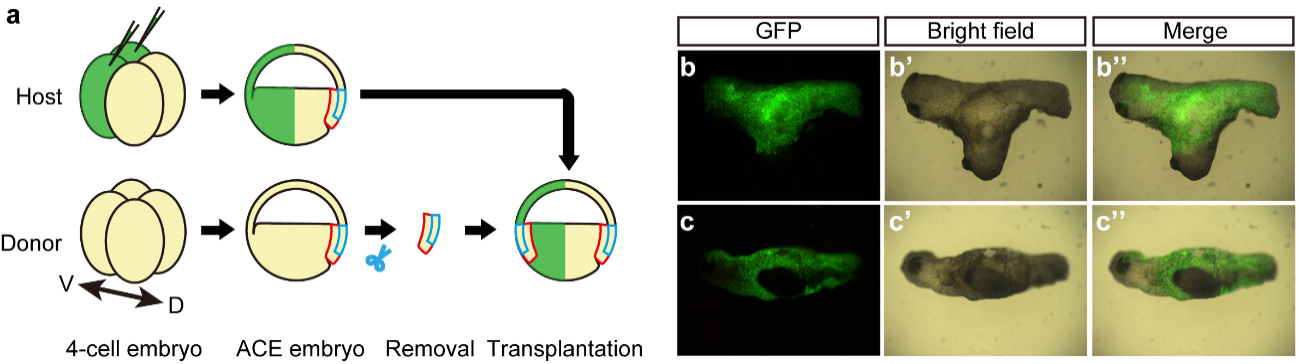


**Supplementary Fig. 1 Transplantation of non-labeled dorsal marginal zone at ACE into the ventral side of mGFP-labeled ACE embryo.**

(a) Schematic drawing of the transplantation assay. (b) The lateral view of the operated embryo at tailbud stage. The non-labeled secondary body axis was formed at the ventral side. (c) The ventral view of the operated embryo. The head and the tail of the secondary body axis were completely non-labeled.


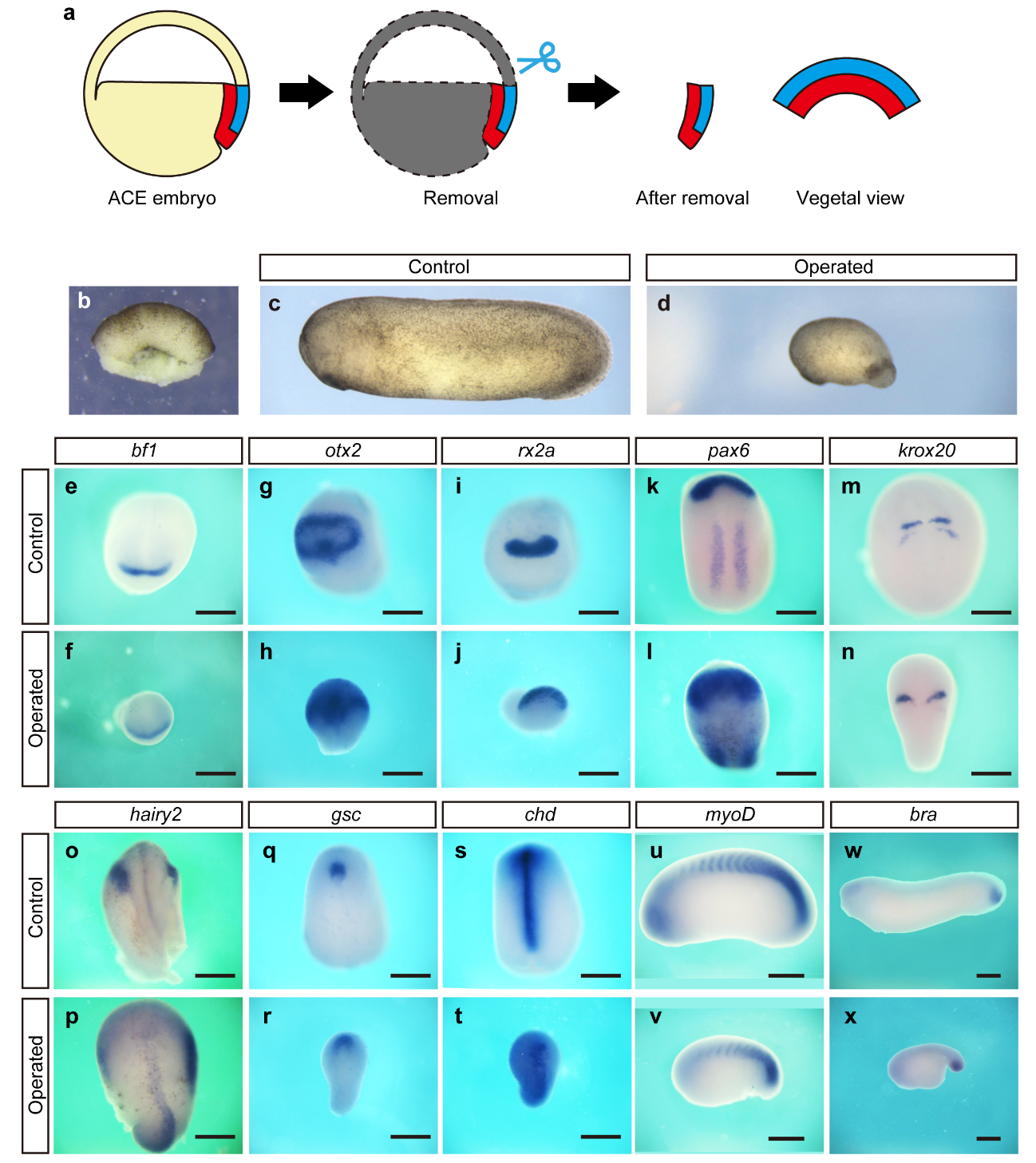


**Supplementary Fig. 2 The explant of dorsal marginal zone at ACE developed into an embryoid possessing a complete dorsal structure**

(a) Schematic drawing of dorsal marginal zone explant at ACE. (b) The explant of dorsal marginal zone at ACE. (c, d) The tailbud embryos developed from control and operated explant. Scale bars, 1 mm. (e-x) *in situ* hybridization of control and operated explant. Scale bars, 500 μm. The expression of *bf1* (e, f), *otx2* (g, h), *rx2a* (i, j), *pax6* (k, l), *krox20* (m, n), *hairy2* (o, p), *gsc* (q, r), *chd* (s, t) in neurula stage. The expression of *myoD* (u, v), *bra* (w, x) in tailbud stage.
